# Parental reports of hospital- and community-based follow-up services, self-efficacy, and symptoms of depression a few months after discharge of a prematurely born child

**DOI:** 10.1186/s12889-024-19079-4

**Published:** 2024-06-19

**Authors:** Inger Pauline Landsem, Bjørn Helge Handegård

**Affiliations:** 1 Health Research Faculty, Institute of Health and Caring Sciences, UIT the Arctic University of Norway, Tromsø, Norway; 2 Health Research Faculty, UIT the Arctic University of Norway, RKBU North, Tromsø, Norway

**Keywords:** Preterm infants, Follow-up services, Parental self-efficacy, Parental mental health

## Abstract

**Background:**

Many parents report the transition from hospital to home as challenging after the birth of a preterm-born child. This study investigates parental perceptions of community-based follow-up services after hospital discharge, alterations in parental self-efficacy during the early months at home, the prevalence of depressive symptoms among parents, and the relationship between these factors and both NICU experiences and children’s regulative behaviors.

**Methods:**

In this second phase of a descriptive study, 110 parents returned a digital questionnaire when their child was four months corrected for prematurity. Parents were recruited while hospitalized with their child, in one of eight Norwegian neonatal intensive care units (NICUs). Thus, the study provides insight into follow-up services across a broad geographical range. Parents’ perception of self-efficacy was reported on the Karitane Parenting Confidence Scale, and depressive symptoms were evaluated with the Edinburgh Postnatal Depression Scale (EPDS). Children’s regulative behavior was reported on the 6-month version of the Ages and Stages Questionnaire: Social and Emotional (ASQ: SE). Using SPSS, associations between variables were investigated in multiple regression analysis in addition to descriptive analysis. Additionally, the examination of repeated measures of parental self-efficacy involved the application of linear mixed models.

**Results:**

Parents reported improved perception of self-efficacy from postdischarge to the children’s age of four months (F (1,167) = 1233.2, *p* < 0.001). On average, fathers’ self-efficacy improved more than that of mothers. Parents’ perception of being well informed prior to discharge from hospital predicted improved self-efficacy (F [1, 29] = 10.4, *p* = 0.003). Reports of depressive symptoms were at a similar level as previously reported among new parents, as 10.4% of mothers and 6.7% of fathers reported EPDS scores *≥* 10 points. Parents’ reports on ASQ: SE show that 15% of the children scored above the recommended cutoff score for three- to nine-month-old children. The parent-reported benefit of follow-up services showed considerable variation. The importance of specific knowledge about prematurity among public health nurses and physicians was frequently mentioned, and public health nurses were perceived as coordinators and mediators of various services.

**Conclusions:**

Parents reported improved self-efficacy, and depressive symptoms at similar levels as new parents in general, a few months after discharge from hospital. Childrens’ regulatory behavior were reported at levels comparable with term-born infants.

## Background

Transitions from a neonatal intensive care unit (NICU) to home continue to place a great deal of stress on parents of preterm born children (hereafter named preterm infants) [1, 2]. In the NICU, families are served by specially educated professionals, and children’s health is continually monitored by tests and technical equipment. In addition, these units are currently characterized by family-centered care and parental participation in the daily care of preterm infants. Depending on how the complex services are organized and delivered, they might strengthen parents’ competence and self-efficacy or possibly extend parental feelings of dependency on professional support in the parenting of their child [3].

Several interventions aiming to improve outcomes for preterm infants and their families have been introduced during the last two decades [4]. The most significant effects seem related to interventions that focus on parent‒child closeness (e.g., Kangaroo care) [5], parental involvement in the daily care of preterms [6] and structured guiding programs aiming to support parental understanding of a child’s expressiveness and needs [7–9].

The importance of well-planned follow-up after hospitalization in a NICU has been emphasized [10]. Most parents increase parenting skills and knowledge during the NICU stay, but many report heightened concerns related to their child’s development and growth when they prepare to leave the safe and supportive environment experienced in a NICU [1]. In Norway, several national guidelines describe the criteria for how and by whom postdischarge follow-up of preterm infants should be managed [11, 14]. In this context, postdischarge follow-up refers to all kind of services that a preterm-born child might receive after discharge from a NICU. Some are referred to pediatric out-clinic services because of extreme low gestational age at birth or other medical problems, and many preterm-born children are referred to child-physiotherapy. Some NICU’s are offering nurse-led follow-up care of preterms, primarily those in families who can be transferred to home while still dependent of tube feeding and frequent monitoring of weight development. In addition, all newborns in Norway are invited to participate in a community-based follow-up program led by public-health nurses (PHN). The ordinary program consists of ten individual or group-based consultations with each family across the first year after birth. Preterm-born children are enrolled in the same program as term-born children [12–14].

A review of this research area reveals that studies monitoring parental satisfaction with follow-up services in this transitional phase are rare. The first paper from the current study reported on parents’ experiences from their NICU stay [15]. The present study investigates parents’ satisfaction with community-based follow-up services, the development of self-efficacy across the first month at home, and scope of depressive symptoms when their preterm infant was four months, corrected for prematurity. Associations between these factors and NICU experiences and the childrens regulatory behavior are investigated. This might expand our knowledge about and understanding of how services, at different organizational levels, enables to support families of preterm infants in a vulnerable transition phase.

The study aims were as follows:

1. To assess parental satisfaction regarding community-based follow-up services during the initial months following hospital discharge.
2. To examine the development of parental self-efficacy from the second week after discharge until their child reaches four months, adjusted for prematurity.
3. To examine the scope of parents’ depressive symptoms, and explore the associations between the level of problems and previously reported NICU factors and aspects of follow-up services.
4. To explore the associations between parents’ reports of their children’s development and previously reported NICU factors, aspects of follow-up services, and parental mental health.

## Methods and material

This paper presents findings from the second part of a descriptive and explorative study performed in 2019. Here, parents reported on experiences with postdischarge services, their perception of self-efficacy, symptoms of depression and regulative behavior of their child at four months corrected age.

Initially, 14 of the largest Norwegian NICU’s were formally invited to participate in the recruitment of informants to the study. Eight NICU’s, agreed to be involved, and they varied both in size and patient flow. Two are classified as regional intensive care units, four as intensive care units and two as special care nurseries. The eight NICU’s are geographically spread across the four main health-regions in Norway. After the study had received formal approval from the organizational leaders and the local data protection officers, further contact was arranged between the study-manager and a local research assistant in each unit. All research assistants were nurses in different positions in the NICUs.

Parents (mothers, fathers, or comothers), who were hospitalized with a preterm born infant, (gestational age between 23 and 36 + 5 weeks) in the NICU, were eligible as participants in the study. The local research assistants informed parents about the study and collected their informed written consents approximately one week before the child was discharged from the NICU. In the two largest, regional NICUs some parents were lost to this study because they already were enrolled in other studies. Parents written consents, with information about their e-mail address was send to the study manager. Participants were recruited during a 6-month period (January – June 2019), and 212 parents chose to participate.

**Fig. 1:**
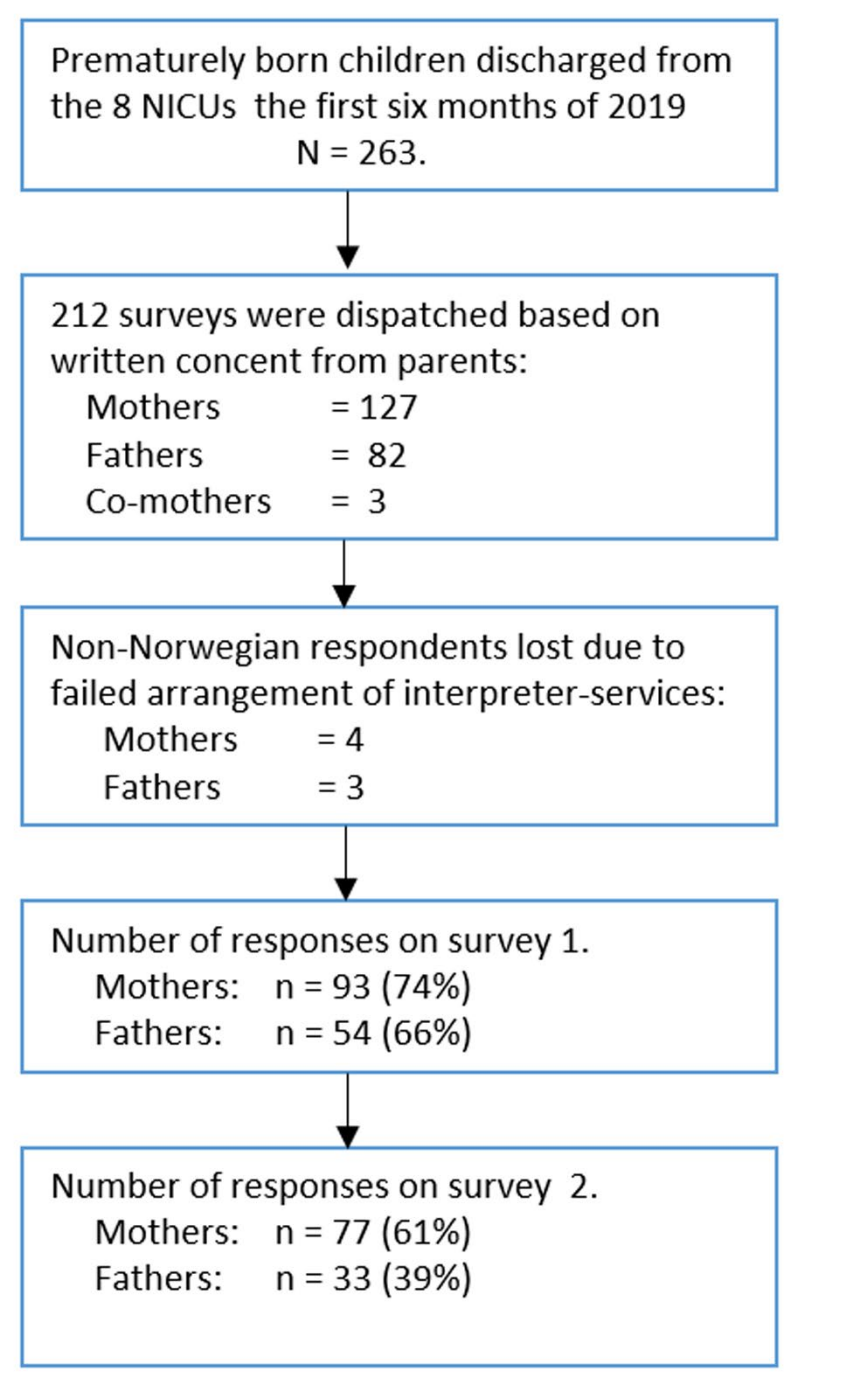
Study participation

This second survey in the study was sent electronically to the 212 participants four months after each child’s term date. An overview of the participants is shown in Fig. 1.

Within families with twins, parents were requested to attribute their responses to the smallest infant. However, the data reveal that some parents opted to provide separate responses for each individual infant.

### Questionnaires

The survey consisted of 32 questions addressing parents’ perception of various follow-up services after discharge, including demographic information and information about possible rehospitalizations. The following questions encompassed three validated questionnaires addressing development and regulatory behavior of the child (Ages & Stages Questionnaire: Social and Emotional), parents’ perception of self-efficacy (The Karitane Parenting Confidence Scale) and a central aspect of parental mental health (Edinburgh Postnatal Depression Scale). In addition, the questionnaire included four open-ended questions where parents could describe what they perceived as (1) the purpose with follow-up home-visits, (2) if their child got follow-up services from others than public-health nurses and doctors, (3) what they might had perceived as valuable in the follow-up and lastly, (4) if there were aspects of support they had missed.

Information about children’s development and regulatory behavior, including problems and resources, was collected using 22 questions from the 6-month version of the “Ages & Stages Questionnaire: Social and Emotional” (ASQ: SE) [16]. The ASQ: SE covers seven dimensions: self-regulation, compliance, communication, adaptability, autonomy, level of emotionality and interaction with caregivers and is recommended for children aged 3 to 9 months [16, 17]. Nineteen of the questions may be used to calculate a sum score. Parents are asked to rate aspects of their child’s behaviors as occurring “most of the time”, “sometimes” or “rarely or never”, which corresponds to scores of 0, 5 and 10 points, respectively. If the behavior causes concern, an additional 5 points are added to the score for that item. The sum score ranges from zero, indicating no problems or concerns, to a maximum score of 285 points if the respondent has maximal problems and concerns on all questions. The 6-month version of the ASQ-SE questionnaire has a cutoff score of 45. Total scores above 45 are recommended to generate a more intensive follow-up of the child. The instrument is mostly used in face-to-face consultations with caregivers but is also used in surveys [16, 17].

Parents were asked to report their perception of self-efficacy using the Karitane Parenting Confidence Scale (KPCS) in both surveys, first approximately two weeks after discharge and next when their child was four months, corrected for prematurity. The KPCS is a validated instrument that sheds light on three underlying dimensions: parenting, social support, and the infant’s development [18]. The KPCS encompasses 15 statements rated on a Likert scale (0–3), from ‘Hardly ever’ to ‘Almost always’, and a total score between 0 and 45 points can be computed. The KPCS was designed in Australia [18, 19] and was translated into Norwegian for this study by the first author and an English government-authorized translator [15].

Last, this survey addressed parents’ perceptions of their own mental health using the Edinburgh Postnatal Depression Scale (EPDS) [20]. The EPDS is a well-known, validated and frequently used screening instrument consisting of 10 brief questions [20, 21]. The parents are asked to look back on the last seven days and answer on a Likert scale (ranging from 0 to 3) for each of the 10 questions [22]. A total sum score between 0 and 30 points can be calculated.

### Ethical considerations

The study was approved by the Northern Regional Committee for Medical and Health Research Ethics (REK), project number 2018/1948, the data protection officer at the University Hospital of Northern Norway (UNN) (ref. 2019/341) and the data protection officers at the hospitals that recruited participants. An agreement on data storage and use of the data processing tool REDCap was signed with UNN’s Clinical Research Department in January 2019. Participants were informed by healthcare personnel in the respective NICUs, from which the children were discharged, regarding their entitlement to withdraw their consent for participation in the study. In addition, if the questionnaire raised concerns the parent was advised to contact their public health center.

### Data collection and analysis

Parents’ email addresses were transferred to the REDCap system by the project manager, and the second questionnaire was dispatched at children’s age of four months, corrected for prematurity. In cases where parental responses on the questionnaire were delayed, reminders were generated to prompt their participation.

Data were analyzed using IBM SPSS, version 26. In addition to descriptive and correlational analyses, an analysis of parents’ change in self-efficacy (KPCS) was performed using linear mixed model (LMM) analysis, correcting for the clustering effect of twin pairs. Associations between parental reports on their own mental health (EPDS) and child-, demographic- and NICU-related factors were investigated using multiple regression analysis.

## Results

One hundred and ten parents responded on the second survey, 40 less than on the first survey [19]. Birth or demographic variables were altered to a small extent because of the reduced sample size (Table 1).

**Table 1:** Child and demographic information

Child-related information**	1. survey		2. survey	
Birth weight: n (mean/SD)	*N* = 111 (1939 g/738 g)		*N* = 87 (1942 g/734 g)	
Gestational age: (mean)	32 weeks and 6 days		32 weeks and 4 days	
Gender (n): (boys/girls/unknown)	(62/47/2)		(46/39/2)	
Pairs of twins:	24		19	
Respiratory support (CPAP/HFNC: n (% of N), mean days	76 (68%), 16.1		62 (71%), 4.0	
Mechanical ventilation: n (% of N), mean days	21 (19%), 7.1		20 (23%), 1.1	
**Parent-related information**	**Mothers**		**Fathers**	
	1. survey	2. survey	1. survey	2. survey
Participants: n (%) *	96 (74)	77 (61)	54 (66)	33 (39)
Mean age:	30.9	30.7	33.5	33.4
Living with coparent: n (%)	90 (94)	73 (97)	54 (100)	33 (100)
First-time parents: n (%)	41 (43)	33 (45)	30 (57)	19 (66)
Education *≥* 4 years at college/university: n (%)	61 (64)	24 (31)	28 (52)	7 [23]
Born outside Scandinavia: n (%)	11 [12]	8 [10]	3 [6]	0

*Percentage of those who returned a written consent to participate.

**For twins, if the mother and father reported on one child each, both children are included here.

### Parents’ experiences with postdischarge follow-up services

The responses to some of the predefined questions are summarized in Table 2. Most mothers and fathers reported that the first home visit by a public health nurse (PHN) took place within the first week after discharge from the NICU, with a mean of 7.8 days (median 5 days). Approximately 60% of families received one home visit, and 25% reported 2 to 4 PHN-home visits. Many families visited a public health center (PHC) frequently the first months after NICU discharge. Five or more visits at the PHC were reported by 59% of the families (*n* = 49), while three to four visits were reported by 37.3% (*n* = 31) of the families.

**Table 2:** Parental satisfaction with community-based follow-up services

Questions about satisfaction with post-discharge follow-up	Respondents	To large/very large extent	To some extent	None or small extent
The PHN* was well informed about my child’s health and situation % (n)	Mothers (75)	58.7% (44)	28.0% (21)	13.3% (10)
	Fathers (32)	50.1% (16)	31.3% (10)	18.8% (6)
The PHN was interested in my child’s unique history % (n)	Mothers (75)	70.7% (53)	24.0% (18)	5.3% (4)
	Fathers (31)	80.7% (25)	6.5% (2)	12.9% (4)
I feel confident with the competence demonstrated by the PHN % (n)	Mothers (74)	52.7% (39)	29.7% (22)	17.6% (13)
	Fathers (32)	62.5% (20)	25.0% (8)	12.5% (4)
I feel confident with the competence demonstrated by the PCP* % (n)	Mothers (74)	54.1% (40)	28.4% (21)	17.6% (13)
	Fathers (32)	56.2% (18)	21.9% (7)	21.9% (7)
PHN-support has been important for the establishment of infant-sleep routines % (n)	Mothers (75)	17.3% (13)	29.3% (22)	53.4% (40)
	Fathers (32)	18.8% (6)	18.8% (6)	62.5% (20)
PHN-support has been important for the establishment of feeding patterns % (n)	Mothers (75)	24.7% (17)	36.0% (27)	41.3% (31)
	Fathers (32)	25.0% (8)	18.8% (6)	56.3% (18)
PHN-support has been helpful in the establishment of social interactions with the child. % (n)	Mothers (75)	30.7% (23)	38.7% (29)	30.7% (23)
	Fathers (32)	37.5% (12)	31.3% (10)	31.3% (10)
Has the PHN-support helped you to get in contact with other health services when needed? % (n)	Mothers (73)	68.8% (48)	16.4% (12)	17.8% (13)
	Fathers (31)	61.4% (19)	19.4% (6)	19.4% (6)

*PCP: public center physician

In open-ended questions, parents were asked about experiences of services that they appreciated or services they missed in the postdischarge follow-up. Twenty-six children (30%) received physiotherapy after discharge from the hospital. The number of preterm infants receiving physiotherapy might be higher as many parents were followed by multiprofessional teams at their hospital. Seven parents responded that the follow-up by a physiotherapist had been especially important, while others emphasized the utility of meeting PHNs that had broad knowledge about preterms and the special needs these families might experience. On the other hand, lack of knowledge about prematurely born children among PHNs and physicians at public health centers was the most frequent response to the question addressing what parents missed in the follow-up of their child.

Eleven families (10%) received home visits by NICU professionals in the transition phase from hospital to home, and 12 families (10.9%) reported that their child had been readmitted to a hospital after discharge. Having received home visits in the transition phase was not correlated with whether the child had been readmitted.

### Parents’ perception of self-efficacy

Mothers and fathers reported quite similar and higher KPCS scores at four months (mean = 40.0 (SD = 3.0)) compared to the postdischarge response (KPCS mean = 25.5 (SD = 3.7)). On average, parent-reported self-efficacy improved with 15 KPCS points (F (1,167) = 1233.2, *p* < 0.001). The increased self-efficacy was positively associated with parents’ report of satisfaction with predischarge information in the first survey (F (1,79) = 13.03, *p* < 0.001) but not with child or sociodemographic factors. While maternal reports of the first to the second measurement of KPCS were significantly and positively correlated (*r* = 0.32, *n* = 76, *p* = 0.006), a nonsignificant association was detected in paternal reports (*r* = 0.24, *n* = 30, *p* = 0.24). Scatterplot investigation indicates that some fathers who reported low KPCS scores in the first survey reported a highly improved sense of self-efficacy in the second survey.

### Parents’ self-report of depressive symptoms four months after the term date

Mothers (*n* = 75) reported a mean EPDS score of 4.3 (SD = 3.6), median = 4.0, and fathers (*n* = 30) reported a mean score of 3.8 (SD = 3.4), median = 3.0. Eight mothers (10.4%) and two fathers (6.7%) reported EPDS scores *≥* 10. Possible associations with other conditions were investigated in multiple regression analysis. No parent- or follow-up variables were significantly associated with the EPDS scores except that higher levels of parents’ perception of support from family and friends while hospitalized in the NICU were associated with lower EPDS scores (t = − 2.80, *p* = 0.006).

### Child developmental outcomes at 4 months corrected age

Mothers (*n* = 77) reported a mean-score on ASQ: SE = 24.62 (SD = 17.46) and fathers (*n* = 33) similarly ASQ: SE = 26.64 (SD = 19.14). A sum score of 45 or above was reported on 13 of 87 children (15%), representing the sample above the recommended cutoff score for 6-month-old children [18]. Only 13 preterm infants were scored above the ASQ: SE cut-off and this results in low statistical power, but analysis show that they didn’t differ significantly from the rest of the sample in relation to birthweight, gestational age, duration of hospitalization or ventilatory support. The ASQ: SE sum scores in maternal reports correlated with some maternal responses from the first survey: whether or not the child had received mechanical ventilation *r* = 0.26, *n* = 75, *p* = 0.024) and the following maternal experiences while living in the NICU: the child had been interested in the mothers face (Spearman’s rho = − 0.26, *n* = 74, *p* = 0.028); the child had responded to the mothers voice (rho = − 0.31, *n* = 74, *p* = 0.006); the most common state when the child was awake (rho = 0.29, *n* = 73, *p* = 0.012) and support from friends and family (rho = − 0.30, *n* = 74, *p* = 0.009). No significant correlations were detected in relation to paternal scores on ASQ: SE. A moderate association between child and parental wellbeing was found, as EPDS and ASQ: SE scores were significantly correlated (*r* = 0.52, *n* = 106, *p* < 0.001).

## Discussion

The responses from parents of preterm infants reported in this study enlighten how these families try to adapt to many different challenges after discharge from a NICU. The main finding is that both mothers and fathers report higher levels of self-efficacy from the early discharge period until approximately four months later. Another important finding is that parents in this sample report the amount of depressive symptoms at similar levels as reported in prevalence studies on new parents in general [22].

The study shows that some families continue to be supported by NICU follow-up services in the transition to home and that most families are frequent users of public health services in their community. Many families received several home visits by their public health nurse, in addition to frequent visits at the public health clinic. Although Table 2 indicates that a group of parents perceived a lack of competence and interest among the health care actors and low confidence in PHC services, most parents reported these services as very helpful. Interestingly, in these first months after birth, parents report that the PHCs are perceived as an important coordinator that may facilitate contact with nonregular services, such as physiotherapists, family centers, and general practitioners. Some parents reported that the PHN is an important cooperative partner in relation to establishing robust patterns for infant sleep, feeding and social interactions. The answers to questions concerning infant sleep, eating and interactions were highly correlated, indicating that families who found the PHN-competence useful received help in addressing several challenges. This confirms the importance of adequate knowledge about the special needs among preterms in public health clinics. On the other hand, some parents may perceive that this kind of knowledge was already received while hospitalized in the NICU [1, 2].

The significant and important improvement in self-efficacy during the first postdischarge months and the moderate to low levels of mental health problems among parents are promising. These findings could be biased by high withdrawal rates from the first to the second survey, for example, the decrease in the proportion of parents with high educational levels among those who participated in the second survey compared to the first. On the other hand, higher education was found to be associated with a higher perception of self-efficacy in a recently published study [23]. Thus, our finding of improved self-efficacy seems quite robust.

Parental self-efficacy refers to individual expectations and beliefs regarding one’s capacity to carry out the parental role skillfully and efficiently [24]. Self-efficacy encompasses both the parental experience of having the knowledge to fulfill their parental role and a feeling of confidence about being able to handle it. Previous research indicates that parents of preterm children are more distressed and feel less confident in their parenting than parents of full-term children [25, 26], and some have suggested the birth of a preterm-born child as potentially traumatic for parents [27]. The current finding of a relatively rapid increase in KPCS scores can be viewed as promising. It might also relate to previous descriptions of higher levels of personal growth among parents of preterm infants compared to those of full-term infants in infancy [28]. Taubman - Ben-Ari et al. (2014) relate their findings to parental support systems and emphasize the importance of building routine professional guidance for new parents [28].

The complexity of supportive actions that parents describe in the current survey may illustrate that both in-hospital and community services designed for families with preterms have become more targeted in Norway. Parents report that different follow-up services collaborate in flexible ways to meet the unique needs within each family. In addition, the NICUs that recruited participants to this study have implemented various strategies to promote parental presence, skin-to-skin contact and partnership in the care of each child [15]. Taken together, these findings may be signs of general improvements in the longitudinal care of preterms and their families, addressing previous calls for such enhancements [2, 10].

Parental reports on the ASQ: SE provide insight into how demanding parents experience the children’s behavior and parenting of the child after some months at home. The 19 questions address aspects of child behaviors in relation to sleeping, eating, social interactions and to what degree the infant is beginning to display self-regulative behavior. ASQ: SE scores should be interpreted with caution, as we lack data to compare with on children at this early age [17]. However, an interesting finding is that maternal reports of more child-state stability and responsive interactive behavior before discharge from the NICU are significantly correlated with lower ASQ: SE sum scores at four months. The most significant correlation appeared between high ASQ: SE scores and infants described by mothers as “constantly shifting states while awake” [15]. This information may be important to consider for clinicians that plan support and follow-up of individual families. Parenting a newborn child who frequently, and often in an unpredictable manner, alternates between sleep and awake states may be very challenging, and more frequent state changes are a known risk factor for developmental challenges [29]. We found a positive correlation between ASQ-scores above cut-off and maternal report on EPDS in the data and this might indicate that challenging transactions in everyday life affect both mothers and children.

## Strengths and limitations

The strength of this survey is the use of several standardized questionnaires and, to some degree, repeated measures [16–18, 20]. In addition, the parents that reported had been discharged from eight different NICUs in Norway; thus, the information is not limited to reports from a single area. On the other hand, this is a small-scale study, and many respondents did not answer the second wave of the study. In addition, many questions in the survey are related to focus addressed in the first phase of this study. Another limitation is that we lack information about parents’ previous health conditions. Thus, this study does not cover all important aspects of follow-up services of preterm born children. The study is descriptive, and the results are to some degree based on simple correlations. The sample was too small to control for twins in statistical analysis. Thus, it only gives a snapshot into a complex field of knowledge. In addition, the data collection took place prior to the pandemic, and it is possible that different results would be obtained if the study was to be placed later in time.

## Conclusions

In this, relatively small descriptive study, parents reported improved self-efficacy and depressive symptoms at similar levels as new parents in general, a few months after discharge of a preterm born child from one of eight participating NICUs in Norway. The report of improved self-efficacy, from post-discharge to approximately four months later, was largest among fathers. Both mothers and fathers reported a positive association between satisfaction with the pre-discharge information received in the NICU and self-efficacy some months later. Parents reported the development of regulatory behavior in their child at levels comparable with term-born infants. Parent-reported satisfaction with community-based follow-up services showed considerable variation. Many parents reported more frequent contact with community-based follow-up services than described as standard follow-up in national guidelines, and many described the public health nurse as an important provider and coordinator of services.

## Data Availability

The dataset used and analysed during the current study is available from the corresponding author on reasonable request.
